# Use of two-point and six-point Dixon MRI for fat fraction analysis in the lumbar vertebral bodies and paraspinal muscles in healthy dogs: comparison with magnetic resonance spectroscopy

**DOI:** 10.3389/fvets.2024.1412552

**Published:** 2024-09-25

**Authors:** Hye-Won Lee, Ji-Yun Lee, Joo-Young Lee, Seung-Man Yu, Kija Lee, Sang-Kwon Lee

**Affiliations:** ^1^Department of Veterinary Medical Imaging, College of Veterinary Medicine, Kyungpook National University, Daegu, Republic of Korea; ^2^Department of Radiological Science, College of Medical Sciences, Jeonju University, Jeonju, Republic of Korea

**Keywords:** canine, chemical shift, intervertebral disc disease, magnetic resonance imaging, myosteatosis

## Abstract

**Introduction:**

Fatty degeneration of the vertebral bodies and paravertebral muscles is associated with the presence, severity, and prognosis of spinal disease such as intervertebral disc degeneration. Therefore, the fat fraction (FF) of the vertebral bodies and paraspinal muscles has been considered a potential biomarker for assessing the pathophysiology, progression, and treatment response of spinal disease. Magnetic resonance spectroscopy (MRS) is considered the reference standard for fat quantification; however, it has limitations of a long acquisition time and is technically demanding. Chemical shift-encoding water-fat imaging, called the Dixon method, has recently been applied for rapid fat quantification with high spatial resolution. However, the Dixon method has not been validated in veterinary medicine, and we hypothesized that the Dixon method would provide a comparable assessment of the FF to MRS but would be faster and easier to implement in dogs.

**Methods:**

In this prospective study, we assessed the FF of the lumbar vertebral bodies and paravertebral muscles from the first to sixth lumbar vertebrae using MRS, the two-point Dixon method (LAVA-FLEX), and the six-point Dixon method (IDEAL-IQ) and compared these techniques.

**Results and discussion:**

The FFs of vertebral bodies and paravertebral muscles derived from LAVA-FLEX and IDEAL-IQ showed significant correlations and agreement with those obtained with MRS. In particular, the FFs obtained with IDEAL-IQ showed higher correlations and better agreement with those obtained with MRS than those derived by LAVA-FLEX. Both Dixon methods showed excellent intra- and interobserver reproducibility for FF analysis of the vertebral bodies and paraspinal muscles. However, the test–retest repeatability of vertebral body and paraspinal muscle FF analysis was low for all three sequences, especially for the paraspinal muscles. The results of this study showed that LAVA-FLEX and IDEAL-IQ have high reproducibility and that their findings were highly correlated with the FFs of the lumber vertebral bodies and paraspinal muscles determined by MRS in dogs. The FF analysis could be performed much more easily and quickly using LAVA-FLEX and IDEAL-IQ than using MRS. In conclusion, LAVA-FLEX and IDEAL-IQ can be used as routine procedures in spinal magnetic resonance imaging in dogs for FF analysis of the vertebral bodies and paraspinal muscles.

## 1 Introduction

Intervertebral disc disease (IVDD) is a common spinal disease in dogs and probably the most common condition for performing spinal magnetic resonance imaging (MRI) in dogs (1). Although the mechanism of IVDD has not been elucidated, fatty infiltration of the vertebral bodies and paravertebral muscles May have a significant effect on the onset, progression, severity, and prognosis of IVDD (2–7). In humans, in addition to evaluating the disc and spinal cord, evaluation of fatty degeneration of the spine and paravertebral muscles using MRI has been widely performed for various spinal diseases including IVDD (8, 9). Those studies have revealed that fat quantification using MRI is correlated with histological examination findings and is associated with presence, severity, and prognosis of spinal disease.

In canine spinal MRI, assessment primarily focuses on disc degeneration, herniation, and spinal cord changes to diagnose IVDD. A few studies on fat assessment of the vertebral bodies and paraspinal muscles in dogs with IVDD have been conducted (4–7, 10, 11). Some studies have evaluated changes in the paravertebral muscles using cross-sectional areas of these muscles on computed tomography, but this method is time consuming and nonspecific for fatty degeneration (10, 11). Other studies have used MRI for assessing fatty degeneration of the paraspinal muscles. However, most studies conducted qualitative or quantitative evaluations based on cross-sectional areas of muscles or the signal changes on T1-weighted or short tau inversion recovery images, which is subjective and difficult when evaluating for diffuse changes (4–6).

There are two primary techniques for fat analysis using MRI in humans including magnetic resonance spectroscopy (MRS) and chemical shift imaging such as the Dixon method. Among them, MRS has been considered the gold standard for fat quantification (12, 13). However, the use of MRS is limited in clinics due to its long acquisition time, technically demanding nature, and associated sampling errors that lead to low spatial resolution (13–15). Chemical shift-encoding water-fat imaging, called the Dixon method, has recently been applied for rapid fat quantification with high spatial resolution (10, 12, 14). It acquires both in-phase (water + fat) and out-phase (water – fat) images and provides a series of four images including in-phase, out-phase, fat-only, and water-only images. The two-point Dixon method was traditionally used, but variants of the Dixon technique such as IDEAL-IQ have been developed to provide more consistent separation of fat and water signals (16, 17).

While several studies have demonstrated the accuracy of the Dixon method and its high clinical applicability for evaluating fat quantification in the spine and paravertebral muscles in humans, research in dogs remains limited (2, 3, 8, 9). To the best of author’s knowledge, only one study has applied the two-point Dixon technique to assess paraspinal muscle myosteatosis in dogs with IVDD. This study revealed an association between myosteatosis in the multifidus muscle and outcomes (7). Because fat quantification MRI can be influenced by various factors such as the composition or size of the field of view, it is crucial to establish the accuracy, reproducibility, and repeatability before applying it in small dogs. The purpose of this study was to evaluate the feasibility of the traditional two-point Dixon method and a more recently developed six-point Dixon method compared with gold standard of MRS for fat fraction (FF) analysis of the vertebral bodies and paravertebral muscles in healthy dogs.

## 2 Materials and method

### 2.1 Animals

This study was a prospective experimental study. In this study, six purpose-bred beagles, including four intact female and two intact male dogs, were used. The median age of the dogs was 3 years (1–5 years), and the median weight was 13.4 kg (12.0–14.8 kg). All dogs were regarded as clinically healthy based on a physical examination, complete blood count, serum biochemistry, and thoracic and abdominal radiographs. The study protocol was authorized by the Institutional Animal Care and Use Committee at Kyungpook National University. The protocol for the care of the dogs adhered to the Guidelines for Animal Experiments of Kyungpook National University (No. KNU 2023–0590). Each laboratory beagle dog was housed in an individual pen and had no history of low back pain, spinal surgery, or metallic implant placement.

### 2.2 MRI acquisition

The dogs were fasted for 24 h prior to anesthesia induction for MRI. A 22-gauge catheter was placed into the cephalic vein, and 0.03 mg/kg of medetomidine (Medetin, 1 mg/mL, Dongbang, Gyeonggi-do, Korea) and 1 mg/kg of alfaxalone (Alfaxan^®^, 10 mg/mL, Careside, Gyeonggi-do, Korea) were administered to each dog. An endotracheal tube was placed, and anesthesia was maintained with isoflurane (Ifran^®^, 2–3%, Hana Pharm, Seoul, Republic of Korea) and oxygen (1–2 L/min). General anesthesia and breathing were controlled with a ventilator. Isotonic saline solution (0.9% NaCl, 3 mL/kg/h, JW Life Science, Seoul, Republic of Korea) was administered intravenously for the duration of the procedure.

All lumbar MRI images were obtained in dorsal recumbency with a 1.5-T MRI (Signa Explorer; GE Healthcare, Wisconsin, United States) using a 16-channel flex coil (GEM flix coil 16-M Array; GE Healthcare). After obtaining three-dimensional T1-weighted (T1W) sequences as a localizer, three-orthogonal T2-weighted (T2W) images covering the 11th thoracic vertebra to the sacrum including sagittal, transverse, and dorsal planes were obtained.

### 2.3 LAVA-FLEX, IDEAL-IQ, and MRS acquisition for FF of the vertebral bodies and paraspinal muscles

Based on the sagittal, transverse, and dorsal plane T2W images, fat quantification scanning was performed with three different sequences including the two-point Dixon method (LAVA-FLEX; GE Healthcare), the six-point Dixon method (IDEAL-IQ; GE Healthcare), and MRS. MRS was used as a reference standard for fat quantification in this study. Detailed parameters for each sequence are shown in Tables 1, 2. For LAVA-FLEX and IDEAL-IQ, both the sagittal plane in the centre of the lumbar spine and the transverse plane at the centre of the lumbar vertebral midbodies from L1–L6 were acquired for evaluating the vertebral bodies and paraspinal muscles, respectively. Both sequences automatically reconstructed fat signal-only, water signal-only, in-phase, and out-phase images. The IDEAL-IQ additionally provided an automatically calculated FF map (fat signal/fat signal + water signal) on the MRI machine workstation (Signa Explorer; GE Healthcare).

**Table 1:** Acquisition parameters of LAVA-FLEX and IDEAL-IQ for FF quantification of the lumbar vertebral bodies and paravertebral muscles.

Imaging parameters	LAVA-FLEX (two-point Dixon)		IDEAL-IQ (six-point Dixon)	
	Vertebra body	Muscle	Vertebra body	Muscle
Orientation	Sagittal	Transverse	Sagittal	Transverse
Repetition time (msec)	6	6	20	23
Echo time (msec)	2.1, 4.2	2.1, 4.2	3.5, 5.5, 7.4, 9.4, 11.4, 13.3	3.4, 5.4, 7.3, 9.3, 11.2, 13.2
Field of view (mm)	280 × 280	280 × 280	280 × 280	280 × 280
Matrix size	160 × 160	160 × 160	140 × 140	140 × 140
Section thickness (mm)	6.0	4.0	6.0	4.0
Intersection gap (mm)	0	0	0	0
No. of sections	40	40	40	40
Flip angle (degree)	12	12	5	5
No. of signals acquired	2	2	8	8
Bandwidth (kHz)	62.5	62.5	100	100
Imaging time (sec)	106	108	155	156

FF; fat fraction.

**Table 2:** Acquisition parameters of MRS for FF quantification of the lumbar vertebral bodies and paravertebral muscles.

Parameters	Vertebral bodies	Paravertebral muscles
Sequence type	Stimulated echo acquisition mode (STEAM)	
Repetition time (msec)	3,000	3,000
Echo time (msec)	35	35
Number of averages	32	32
Acquisition voxel (mm)	4.0 × 6.0 × 15.0	4.0 × 15.0 × 15.0
Number of signals acquisition	8	8
Imaging time (sec)	168	168

FF; fat fraction. MRS; magnetic resonance spectroscopy.

**Figure 1:**
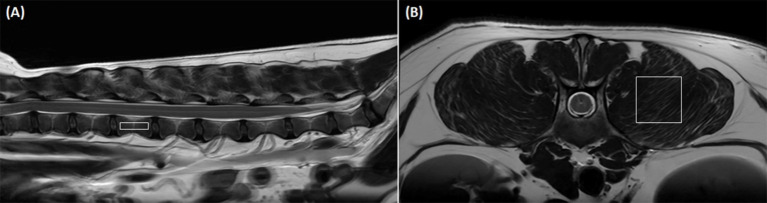
Positioning of the voxel in magnetic resonance spectroscopy (MRS) to analyze the fat fraction (FF) in the lumbar vertebral bodies and paraspinal muscles. **(A)** Shows a representative sagittal image of voxel positioning for vertebral body FF measurement with MRS. A voxel (4.0 × 6.0 × 15.0 mm) is positioned in the center of the lumbar vertebral body without including cortical bone and the paraspinal muscles based on transverse, sagittal, and dorsal images. **(B)** Shows a representative transverse image of voxel positioning for paraspinal muscle FF measurement with MRS. A voxel (4.0 × 15.0 × 15.0 mm) is positioned in the left epaxial muscles at the level of the vertebral midbody without including adjacent subcutaneous muscles based on transverse, sagittal, and dorsal images.

To acquire MRS as a standard of reference, single-voxel stimulated echo acquisition mode (STEAM) was used. Based on the three-orthogonal T2W images, multiple single spectroscopy voxels were positioned in the center of the lumbar vertebral bodies and paraspinal muscles from L1 to L6 (Figure 1). For the vertebral bodies, a voxel size of 4.0 × 6.0 × 15.0 mm was used. This voxel size was set to the largest size that does not include the cortical bone and surrounding muscles to prevent errors on MRS due to low signal-to-noise ratio. For the paraspinal muscles, a voxel was positioned in the left epaxial muscle at the level of each vertebral midbody using a voxel size of 4.0 × 15.0 × 15.0 mm. The voxels of the paravertebral muscles were set to the largest so that all sides of the voxels were surrounded by the muscles without including adjacent subcutaneous fat. Both the longissimus lumborum and iliocostal muscles were included because it was difficult to distinguish the fascial boundaries between these muscles in some cases.

### 2.4 FF analysis with LAVA-FLEX and IDEAL-IQ

The LAVA-FLEX and IDEAL-IQ images were analyzed on Picture Archiving and Communication System workstations (INFINITT; Infinitt Healthcare, Seoul, Korea). In LAVA-FLEX images, the FF was measured using fat signal-only images and in-phase images (fat + water signal) as follows: FF = fat signal / (fat + water signal). In IDEAL-IQ, the FF was measured using an automatically calculated FF map.

**Figure 2:**
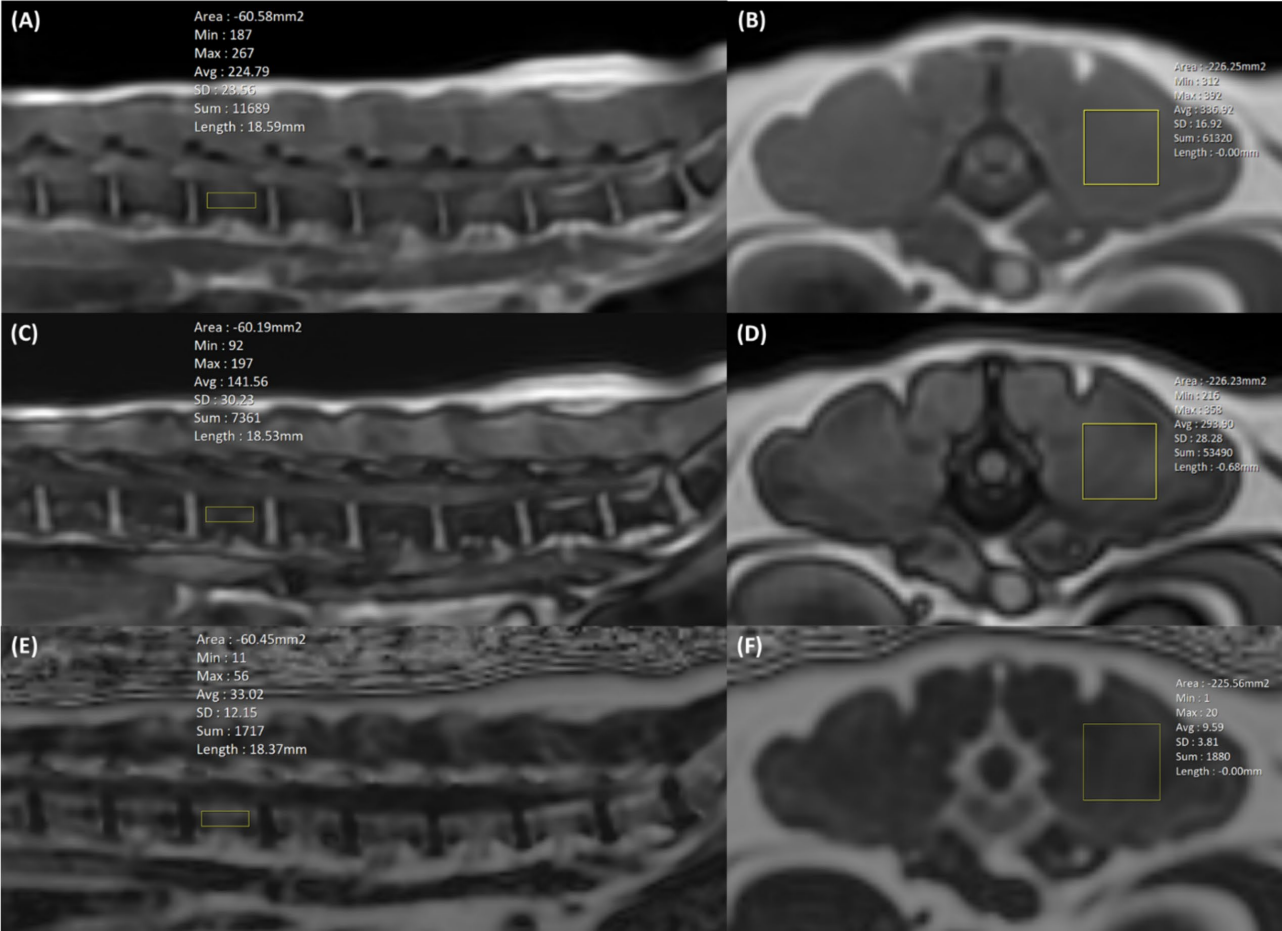
Positioning of the region of interest (ROI) in LAVA-FLEX **(A–D)** and IDEAL-IQ **(E,F)** to analyze the fat fraction (FF) in the lumbar vertebral bodies and paraspinal muscles. In LAVA-FLEX, the same two ROIs were used in in-phase **(A,B)** and out-phase images **(C,D)**, and in IDEAL-IQ, one ROI was used in the FF map **(E,F)**. In LAVA-FELX and IDEAL-IQ, a 4.0 × 15.0 mm ROI is positioned in the sagittal plane for vertebral body FF analysis, and a 15.0 × 15.0 mm ROI is positioned in the transverse plane for paraspinal muscle FF analysis. The ROIs of the vertebral bodies and paraspinal muscles were matched as closely as possible to the voxel from magnetic resonance spectroscopy (MRS).

To evaluate the FF of the lumbar vertebral bodies, rectangular regions of interest (ROIs) (4.0 × 15.0 mm) were drawn on sagittal images centred on the lumbar vertebral bodies to exclude the cortical bone and paraspinal muscle (Figures 2A,C,E). To assess the FF of the paraspinal muscles, rectangular ROIs (15.0 × 15.0 mm) were drawn in the left epaxial muscles where it is surrounded by muscle at the level of each vertebral midbody (Figures 2B,D,F). We recorded the x, y, and z axis information of the MRS voxel range, then when drawing ROIs of the vertebral bodies and paraspinal muscles in LAVA-FLEX and IDEAL-IQ images, the evaluation area of ROIs was made to match the MRS voxels. In LAVA-FLEX, the ROIs drawn on the fat signal-only images were copied and pasted to the in-phase images to create the same ROI.

### 2.5 FF analysis in MRS

All spectroscopy images were processed using a commercial spectroscopy tool (LCModel version 6.3; LCModel Inc., Oakville, Canada), which involves post-processing and quantifications including noise filtering, apodization, baseline, phase correction, signal fitting of the peaks within the acquired spectra, and integration to determine the area under each spectral peak of interest. In the spectra, integrated signal intensity in the spectral regions of 0.5–2.0 ppm was assigned to fat, and 4.0–5.4 ppm was assigned to water. The FF was calculated as the ratio of fat peak areas to the sum of fat peak and water peak areas (14).

### 2.6 Reproducibility and test–retest repeatability test

To assess the test–retest repeatability of FF analysis using LAVA-FLEX, IDEAL-IQ, and MRS, the same experiment protocol was performed again with a one-week interval and analyzed with the same methods as the first examination.

To evaluate the intra- and interobserver reproducibility of the LAVA-FLEX and IDEAL-IQ, one examiner (H-WL) analyzed the LAVA-FLEX and IDEAL-IQ images in the first exam three times, and three different examiners (H-WL, J-YL, J-YL) independently performed an analysis of the LAVA-FLEX and IDEAL-IQ without knowing the MRS results and each other’s results. When measuring reproducibility, the ROI was drawn subjectively using the same ROI criteria without the location information of the MRS voxel; 4.0 × 15.0 mm ROIs were drawn on sagittal images centred on the lumbar vertebral bodies to exclude the cortical bone and paraspinal muscle for vertebral body analysis and 15.0 × 15.0 mm ROIs were drawn in the left epaxial muscles where it is surrounded by muscle at the level of each vertebral midbody. The reproducibility of MRS was not evaluated because MRS was analyzed using the program and did not depend on the examiner.

### 2.7 Statistical analysis

All continuous values are reported as means ± standard deviations (SDs). All statistical calculations were performed using IBM SPSS Statistics (SPSS 25.0, IBM SPSS statistics, New York, United States). Normality was assessed using the Kolmogorov–Smirnov test. In the analysis, the comparison between MRS and Dixon used Dixon data where ROIs were drawn consistent with MRS voxels based on MRS location information. To evaluate the difference in the FFs of the lumbar vertebral bodies and paraspinal muscles derived from MRS according to the sites and the differences among MRS, LAVA-FLEX, and IDEAL-IQ, the Friedman test and Wilcoxon signed-rank test were used. To assess the correlation and agreement between LAVA-FLEX and IDEAL-IQ and the reference standard of MRS, Spearman’s correlation, linear regression, and the intraclass correlation coefficient (ICC) analyses were performed. It was considered statically significant if the *p*-value was less than 0.05 (*p* < 0.05). The following criteria were used to analyze the ICC: excellent (≥0.90), good (= 0.75 to 0.89), fair (0.50 to 0.74), and poor (<0.50) (1). The difference between each sequence was analyzed using Bland–Altman analysis.

The intra- and interobserver reproducibility of LAVA-FLEX and IDEAL-IQ were evaluated using the results of the first examination by calculating the ICC. The test–retest repeatability of fat quantification with MRS, LAVA-FLEX, and IDEAL-IQ was evaluated by assessing the difference in values between two separate scans and calculating the coefficient of variation (CV) and by Bland–Altman analysis. The CV was interpreted according to the following definitions: excellent (<10%), good (10 to 20%), acceptable (21 to 30%), and poor (>30%) (18).

## 3 Results

### 3.1 FFs of the lumbar vertebrae and paraspinal muscles according to each lumbar site

**Figure 3:**
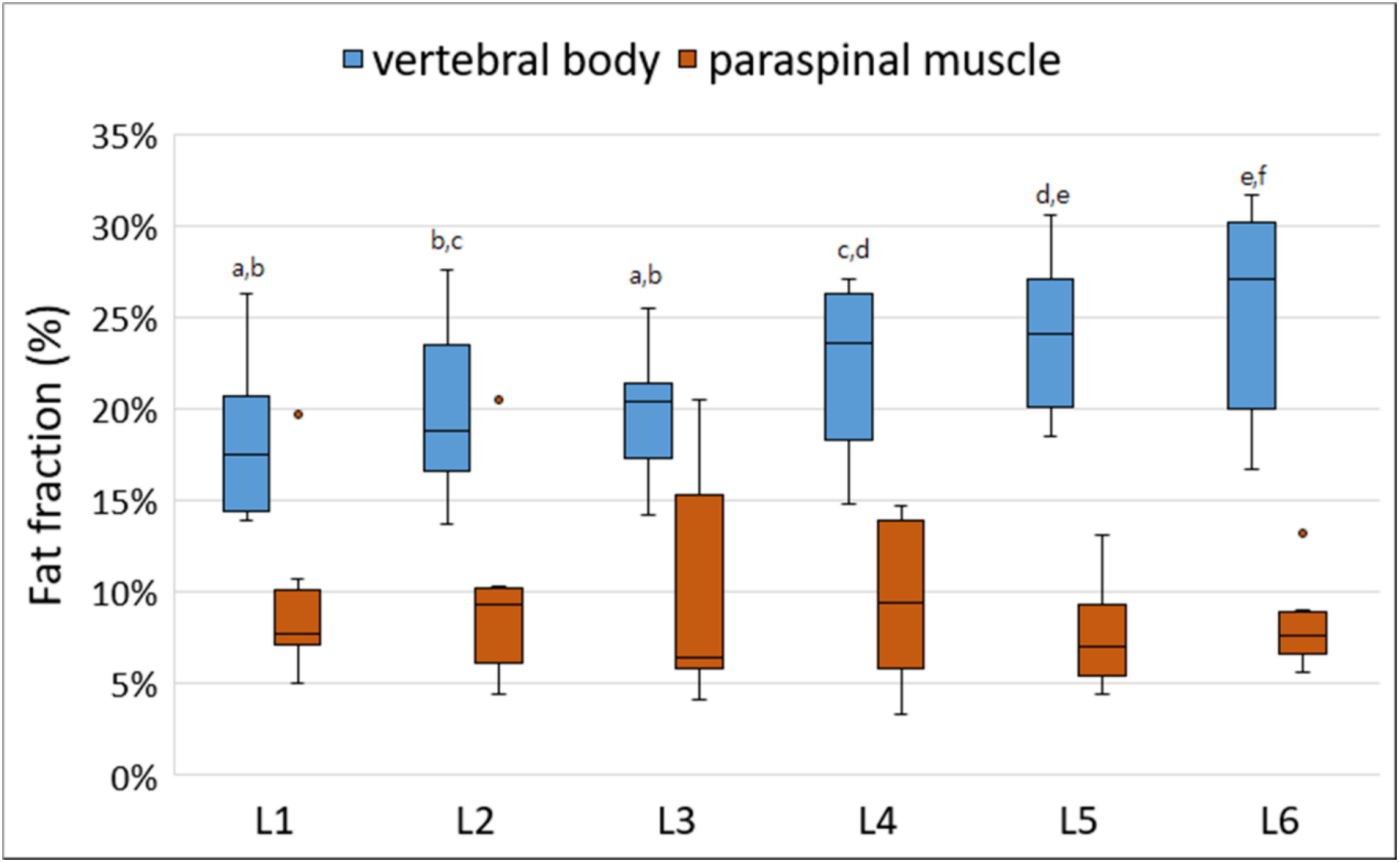
Box-and-whisker plots of fat fraction (FFs) for vertebral bodies and paraspinal muscles derived from magnetic resonance spectroscopy. The boxplot shows the range in data between the 25th and 75th quantiles within the box, line within box indicates median, and outliers are indicated by points. Different lower-case letters, which are at the top of the box plot, indicate significant differences between groups (a–f; *p* < 0.05). The FFs of the lumbar vertebral bodies were significantly different according to the lumbar levels; the FFs of the caudal lumbar spine tended to be higher than those of the cranial lumbar spine. There was no significant difference in the FFs of the paraspinal muscles according to the lumbar levels.

All MRI scans were performed without any complications in all dogs. Figure 3 shows the FF of the lumbar vertebral bodies and paraspinal muscles at each lumbar site derived from MRS. The FFs of the lumbar vertebral bodies were significantly different between the sites; the FFs of the caudal lumbar spine tended to be higher than those of the cranial lumbar spine (Table 3). There was no significant difference in the FFs of the paraspinal muscles according to the sites.

**Table 3:** Fat fracction of the lumbar vertebral bodies and paraspinal muscles derived from MRS according to each lumbar site.

Fat fraction (%)	Lumbar site						*P*-value
	L1	L2	L3	L4	L5	L6	
Vertebral body	18.41 ± 4.85^a^	20.01 ± 5.26^b,c^	19.77 ± 3.99^a,b^	22.15 ± 5.17^c,d^	24.03 ± 4.84^d,e^	25.25 ± 6.42^e,f^	< 0.001
Paraspinal muscle	9.67 ± 5.24	9.84 ± 5.76	10.23 ± 7.11	9.49 ± 4.92	7.78 ± 3.26	8.29 ± 2.75	0.470

The data shown are reported as the mean ± standard deviation. ^a–f^ Within a column, values with different superscript letters differ significantly (*p* < 0.05). FF, fat fraction; MRS, magnetic resonance spectroscopy.

### 3.2 Comparison between MRS, LAVA-FLEX, and IDEAL-IQ

**Figure 4:**
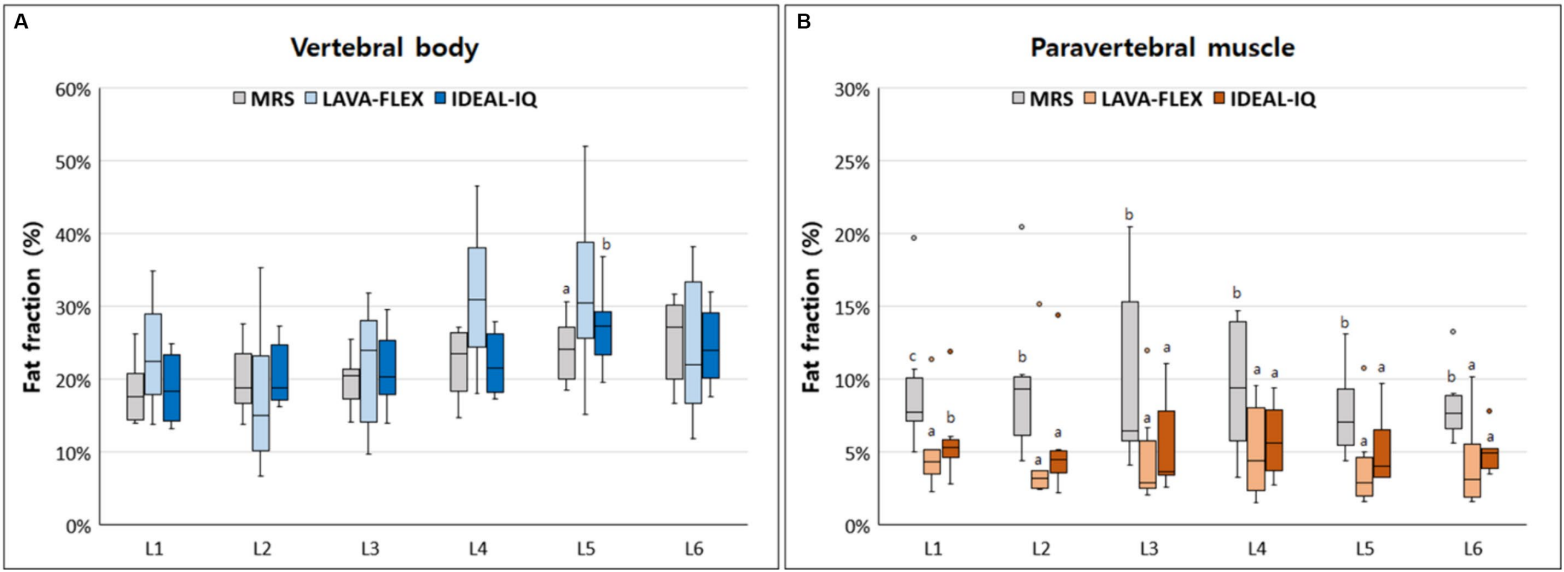
Box-and-whisker plots of fat fractions for **(A)** vertebral bodies and **(B)** paraspinal muscles derived from magnetic resonance spectroscopy (MRS), LAVA-FLEX and IDEAL-IQ. The boxplot shows the range in data between the 25th and 75th quantiles within the box, line within box indicates median, and outliers are indicated by points. Different lower-case letters, which are at the top of the box plot, indicate significant differences between groups (a, b, c; *p* < 0.05).

Figure 4 shows FFs of the lumbar vertebral bodies and paraspinal muscles at each lumbar site derived from MRS, LAVA-FLEX, and IDEAL-IQ. For the FFs of the vertebral bodies, there was no significant difference among the three sequences except for the FF of the 5th lumbar vertebral body (Table 4). The average FF of the vertebral bodies was significantly different between sequences, showing highest value with LAVA-FLEX and the lowest value with MRS. The paraspinal muscle FFs were significantly different between sequences at all sites; they were the lowest with LAVA-FLEX and the highest with MRS. The average FFs of the paraspinal muscles showed the same pattern.

**Table 4:** Comparison among LAVA-FLEX, IDEAL-IQ, and MRS for FF quantification of the lumbar vertebral bodies and paraspinal muscles.

Fat fraction (%)	Site	MRS	LAVA-FLEX	IDEAL-IQ	*P*-value
Vertebral body	L1	18.41 ± 4.85	23.53 ± 8.11	18.80 ± 5.33	0.135
	L2	20.01 ± 5.26	17.75 ± 10.86	20.77 ± 4.90	0.846
	L3	19.77 ± 3.39	21.64 ± 9.13	21.41 ± 5.84	0.607
	L4	22.15 ± 5.17	31.52 ± 10.82	22.18 ± 4.68	0.223
	L5	24.03 ± 4.84^a^	32.26 ± 12.90^a^	27.18 ± 6.05^b^	0.042
	L6	25.25 ± 6.42	24.37 ± 10.88	24.54 ± 5.86	0.846
	Average	21.60 ± 5.36^a^	25.18 ± 11.10^b^	22.48 ± 5.76^b^	0.021
Paraspinal muscle	L1	9.67 ± 5.24^c^	5.16 ± 3.22^a^	5.97 ± 3.13^b^	0.002
	L2	9.84 ± 5.76^b^	5.04 ± 4.98^a^	5.70 ± 4.39^a^	0.009
	L3	10.23 ± 7.11^b^	4.81 ± 3.90^a^	5.57 ± 3.59^a^	0.006
	L4	9.49 ± 4.92^b^	5.13 ± 3.48^a^	5.85 ± 2.72^a^	0.009
	L5	7.78 ± 3.26^b^	4.18 ± 3.47^a^	5.24 ± 2.66^a^	0.042
	L6	8.29 ± 2.75^b^	4.34 ± 3.33 ^a^	5.00 ± 1.58^a^	0.009
	Average	9.22 ± 4.77^c^	4.77 ± 3.52^a^	5.55 ± 2.92^b^	<0.001

The data shown are reported as the mean ± standard deviation. ^a–c^ Within a column, values with different superscript letters differ significantly (*p* < 0.05). FF; fat fraction. MRS; magnetic resonance spectroscopy.

**Figure 5:**
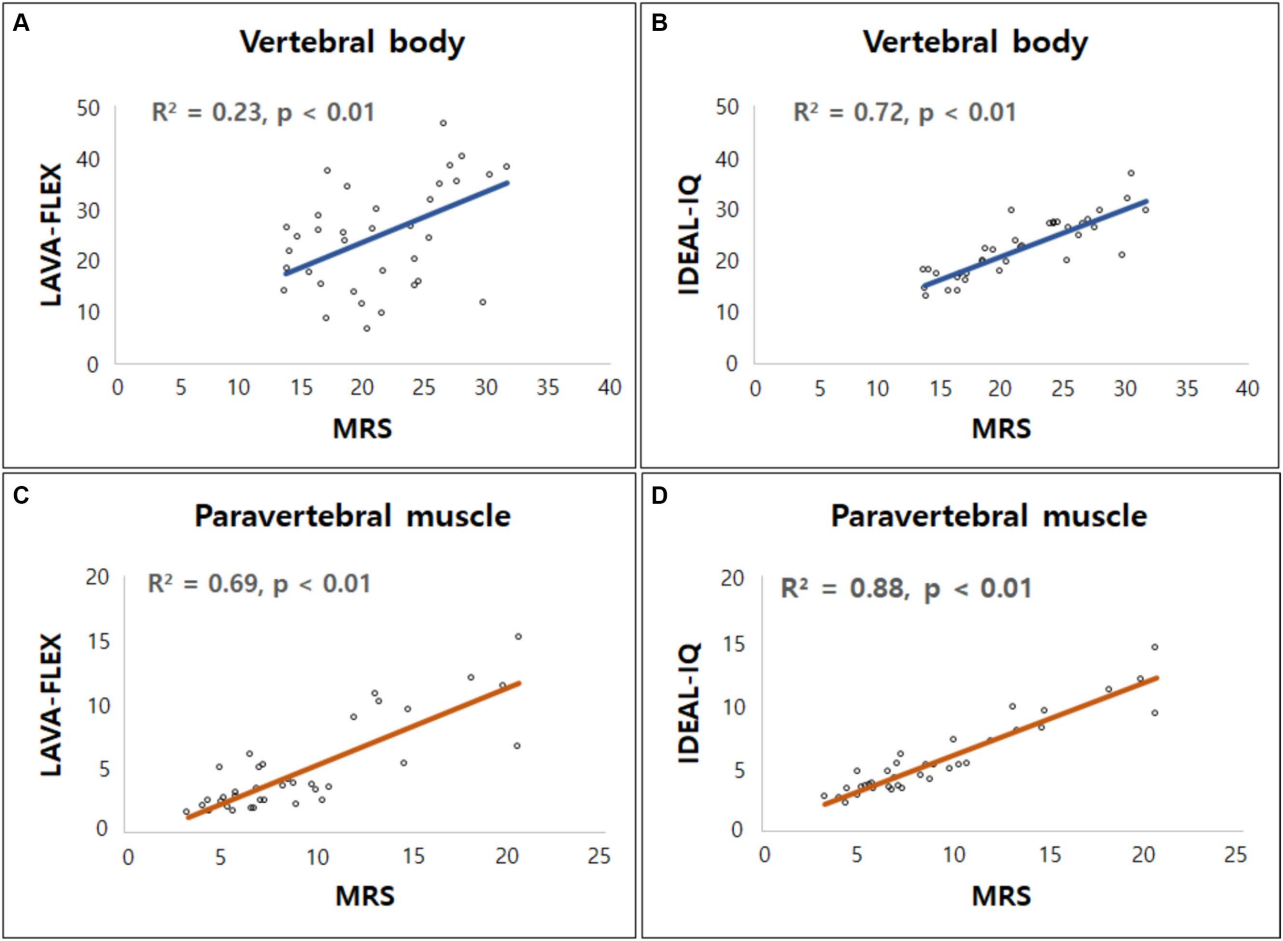
Linear regression model for fat fractions (FF) obtained from Dixon MRI (LAVA-FLEX and IDEAL-IQ) and magnetic resonance spectroscopy (MRS). Scatterplots correlate FFs derived from **(A)** LAVA-FLEX, **(B)** IDEAL-IQ for vertebral body, and **(C)** LAVA-FLEX, **(D)** IDEAL-IQ for paraspinal muscle along the y-axis with fat fraction measured with MRS along the x-axis. Correlation coefficient (R^2^) was ranging from 0.23 to 0.69 for LAVA-FLEX and 0.72 to 0.88 for IDEAL-IQ.

Although the absolute FFs of the vertebral bodies and paraspinal muscles did not match between the sequences, there was a significant correlation between each sequence. The correlation and agreement between LAVA-FLEX and IDEAL-IQ compared with MRS are shown in Table 5. IDEAL-IQ showed higher correlation and agreement with MRS for the FFs of the lumbar vertebral bodies and paraspinal muscles than LAVA-FLEX. A linear regression results showed a significant linear relationship between the FFs derived with LAVA-FLEX and MRS and between IDEAL-IQ and MRS (Figure 5).

**Table 5:** Correlation and agreement between LAVA-FLEX and IDEAL-IQ compared with MRS for FF quantification of the lumbar vertebral bodies and paraspinal muscles.

Parameter	Sequences	Correlation	Agreement			Bland–Altman analysis	
		*r*	*P*-value	ICC	*P*-value	Bias	95% LOA
Vertebral body	LAVA-FLEX vs. MRS	0.437	0.008	0.542	< 0.05	−3.6	−22.7 to 15.6
	IDEAL-IQ vs. MRS	0.859	< 0.001	0.915	< 0.01	−0.9	−6.9 to 5.2
Paraspinal muscle	LAVA-FLEX vs. MRS	0.750	< 0.001	0.885	<0.01	4.4	−0.8 to 9.7
	IDEAL-IQ vs. MRS	0.888	< 0.001	0.911	< 0.01	3.7	−0.8 to 8.1

FF, fat fraction; ICC, intraclass correlation coefficient; LOA, limits of agreement; MRS, magnetic resonance spectroscopy.

### 3.3 Reproducibility and test–retest repeatability

Table 6 shows the intra- and interobserver reproducibility of the FF analysis using LAVA-FLEX and IDEAL-IQ. All the FFs showed an excellent inter- and intraobserver reproducibility. Table 7 summarizes the differences, CVs, Bland–Altman analysis, and ICCs of the FFs derived with LAVA-FLEX, IDEAL-IQ and MRS between the first and second scans. The FFs of the vertebral bodies derived from IDEAL-IQ and the FFs of the vertebral bodies and paraspinal muscles derived from MRS showed significant differences between the first and second scans. The general test–retest repeatability was low for all sequences; in particular, the paraspinal muscle FFs showed poor test–retest repeatability.

**Table 6:** Intra- and interobserver reproducibility of FF analysis using LAVA-FLEX and IDEAL-IQ.

Sequence	Parameter	Interobserver reproducibility	Intraobserver reproducibility		
		ICC	95% CI	ICC	95% CI
LAVA-FLEX	Vertebral body	0.994	0.989–0.997	0.975	0.957–0.987
	Paraspinal muscle	0.998	0.996–0.999	0.998	0.997–0.999
IDEAL-IQ	Vertebral body	0.994	0.990–0.997	0.977	0.960–0.988
	Paraspinal muscle	0.999	0.997–0.999	0.999	0.998–0.999

CI, confidence interval; FF, fat fraction; ICC, intraclass correlation coefficient. An ICC < 0.50, between 0.50 and < 0.75, between 0.75 and < 0.90, and ≥ 0.90 was considered poor, fair, good, and excellent measurement reliability, respectively.

**Table 7:** Difference, coefficient of variance, Bland–Altman agreement, and ICC of the vertebral body and paraspinal muscle fat fractions between the first and second scans.

Sequence	Site	Mean ± SD (%)		*p*-value	CV (%)	Bland–Altman Bias (95% LOA)	ICC (95% CI)
		1st scan	2nd scan				
LAVA-FLEX	Vertebral body	25.18 ± 11.10	26.47 ± 8.89	0.293	38.7	−1.29 (−16.31–13.72)	0.830 (0.667–0.913)
	Paraspinal muscle	4.77 ± 3.52	4.19 ± 2.64	0.134	69.2	0.58 (−5.34–6.51)	0.691 (0.394–0.842)
IDEAL-IQ	Vertebral body	22.48 ± 5.76	23.84 ± 5.82	0.000	25.0	−1.36 (−5.03–2.30)	0.973 (0.946–0.986)
	Paraspinal muscle	5.55 ± 2.92	5.37 ± 2.64	0.777	50.7	0.18 (−1.94–2.30)	0.961 (0.923–0.980)
MRS	Vertebral body	21.60 ± 5.36	24.11 ± 4.55	0.000	22.3	−2.51 (−6.73–1.72)	0.951 (0.903–0.975)
	Paraspinal muscle	9.22 ± 4.77	7.97 ± 4.10	0.001	51.9	1.25 (−2.62–5.12)	0.948 (0.898–0.974)

CI, confidence interval; CV, coefficient of variance; ICC, intraclass correlation coefficient; LOA, limits of agreement; MRS, magnetic resonance spectroscopy, SD, standard deviation.

## 4 Discussion

In this study, LAVA-FLEX and IDEAL-IQ showed excellent correlation and agreement with MRS for evaluating the FFs of the lumbar vertebral bodies and paraspinal muscles, which is consistent with the findings of previous human studies (12–15). However, the absolute FFs derived from LAVA-FLEX and IDEAL-IQ did not exactly match those derived from MRS, particularly in the paraspinal muscles. Our results showed that LAVA-FLEX and IDEAL-IQ slightly overestimated the FFs of the vertebral bodies, while they underestimated the FFs of the paravertebral muscles compared with the MRS. Although the exact cause of the difference between LAVA-FLEX or IDEAL-IQ and MRS is unknown, it May be explained by the multiple confounding factors for FF analysis in this study such as T2^*^ effects, magnetic field inhomogeneity, T1 effects, the presence of multiple peaks in the fat spectrum, and eddy current effects (14, 19–23).

Among the aforementioned confounding factors, the T2^*^ effect is an important factor in fat quantification of the vertebral bodies using MRS (14, 23). Vertebral bone marrow is comprised of trabecular bone, which is the principal source of magnetic field inhomogeneities in the vertebral bodies (24, 25). Magnetic field inhomogeneities shorten the T2^*^ relaxation time of both water and fat components, which broadens the widths of water and fat peaks in MRS (14). As the widths of water and fat peaks become wider, they overlap, and the fat adjacent to water peak is obscured by the water, which leads to underestimation of the FF (14). Compared with MRS, the T2^*^ effect from trabecular bone has a negligible contribution to the signal at gradient echo sequences such as LAVA-FLEX and IDEAL-IQ (14). Therefore, the T2^*^ shortening effect of the trabecular bone May have affected the FFs derived from MRS, resulting in differences in the FFs of the lumbar vertebrae among LAVA-FLEX, IDEAL-IQ, and MRS in this study. Similar to our findings, previous human studies have shown that bone marrow FFs derived from MRS without considering T2^*^ effects were lower than those measured by the Dixon technique (14). Unlike the vertebral bodies, it was thought that there was little T2^*^ effect when evaluating the paraspinal muscle FFs using MRS because there no substance with a large T2^*^ effect was present.

With the Dixon technique, such as LAVA-FLEX and IDEAL-IQ, T1 effects can affect FF measurement. Both LAVA-FLEX and IDEAL-IQ use a very short repetition time. The T1 relaxation time of water is significantly slower than that of fat; therefore, the signal of water May deteriorate as it is exposed to repetitive radiofrequency pulse before the signal is fully recovered due to the short repetition time (19, 20). Although efforts are made to compensate for this by using a low flip angle, the T1 relaxation effect May reduce water signal and contribute to overestimation of FFs with LAVA-FLEX or IDEAL-IQ compared with MRS (19, 20). This T1 effect could explain the overestimation of the vertebral body FF with LAVA-FLEX or IDEAL-IQ compared with MRS, but it is difficult to explain underestimation of paravertebral muscle FF in LAVA-FLEX and IDEAL-IQ. Additionally, when comparing LAVA-FLEX and IDEAL-IQ, LAVA-FLEX had a shorter repetition time and a higher flip angle, which would have resulted in a greater T1 effect and higher fat measurement. However, our results did not reflect this. Therefore, in this study, we believed that the T1 effect was not a major factor that contributed to the fat quantification.

In a previous human study, Dixon techniques tended to underestimate the FFs in organs with low FFs (13). In low fat content regions, image noise can have a significant impact on FFs due to a low signal to noise ratio. Therefore, the Dixon technique May have underestimated FFs due to errors from low signal values and image noise at the low FF sites (26). In our study, the FFs of the paraspinal muscles were low (< 10%) compared with those of the lumbar vertebral bodies (> 20%), and the image noise of the paraspinal muscles with LAVA-FLEX or IDEAL-IQ might be a cause of underestimation of the paraspinal muscle FFs compared with those derived from MRS. In this study, an analysis of image quality, such as signal-to-noise, was not conducted, and further studies are needed to evaluate the relationships among signal, noise, and FF value.

Although the absolute FFs derived from LAVA-FLEX or IDEAL-IQ did not completely match with those determined with MRS, LAVA-FLEX and IDEAL-IQ showed high correlation with MRS for both vertebral body and paraspinal muscle FF assessment. In particular, IDEAL-IQ showed a higher correlation and better agreement with MRS than LAVA-FLEX, which was consistent with the findings of human studies (11, 27). IDEAL-IQ, the 6-point Dixon technique, is thought to be more accurate for FF analysis than the 2-point Dixon-based LAVA-FLEX. There are several advantages of the multi-point Dixon technique compared with the traditional 2-point Dixon technique. The multi-point acquisition offers better chances to correct for the T2^*^ effect, magnetic field inhomogeneity, and eddy currents effects. The multi-point acquisition requires a slightly longer repetition time, which increases signal-to-noise ratio and reduces T1 relaxation effects. However, a longer repetition time has the disadvantage of increasing the scan time. In this study, IDEAL-IQ took approximately 50 s longer than LAVA-FLEX. However, considering that both sequences could be acquired in less than 3 min and that IDEAL-IQ provides FF maps to reduce the analysis time, this small scan time difference is thought be negligible in clinical practice.

Both LAVA-FLEX and IDEAL-IQ had excellent intra- and interobserver reproducibility for vertebral body and paraspinal muscle FF evaluation. This means that when repetitive ROIs are drawn using the same criteria, even if the ROIs do not completely match, they have similar values and can be used clinically. IDEAL-IQ automatically provides an FF map; therefore, only one ROI is needed to measure the FF, but LAVA-FLEX requires that two of the same ROIs to be drawn separately on the fat-only and in-phase images. Therefore, we expected that LAVA-FLEX May show lower reproducibility than IDEAL-IQ, but the reproducibility of the two sequences was similar. This was thought to be because in this study, when drawing two ROIs on a fat-only image and an in-phase image with LAVA-FLEX, we copied one ROI and pasted it on the other image. Moreover, we expected that the reproducibility of paraspinal muscle FFs might be lower than that of the vertebral body FFs because the muscles are not square in shape and have unclear landmarks and margins compared with bone; however, the reproducibility of the FFs for the vertebral bodies and muscles were similar. This was thought to have reduced errors in ROI placement in this study. For accurate comparison with MRS, the ROI was set and analyzed based on the MRS voxel when setting the ROI location for the paraspinal muscles in LAVA-FLEX and IDEAL-IQ in this study.

Unlike their high reproducibility, MRS, LAVA-FLEX, and IDEAL-IQ all had low test–retest repeatability for both vertebral body and paraspinal muscle FF assessment. The test–retest repeatability was the lowest for LAVA-FLEX among the sequences. Although, in this study, the same criteria were used for the first and second examination when setting up voxels for MRS or drawing ROIs for LAVA-FLEX and IDEAL-IQ, but there May have been differences in the scan plane, dog’s position, or setting of voxel or ROIs between first and second examination. However, these factors cannot be completely controlled in clinical practice. The paravertebral muscle FF assessment showed lower test–retest repeatability than that for the vertebral bodies, and there are several possible reasons for this finding. In this study, the voxel size of MRS and the analysis ROI in Dixon methods were larger in the paravertebral muscle than the vertebral body, which May have lowered repeatability. Unlike vertebral body, muscle is a flexible tissue that deforms easily between two scans, especially under anesthesia. Thus, repositioning the same voxel placement exactly as it was for the previous scan is more challenging for the paravertebral muscles than for the vertebral bodies. Moreover, the paravertebral muscle FF can shift depending on the shape and orientation of muscle (28). In addition, the anatomical muscle region used in the analysis May have affected the test–retest repeatability. In this study, when assessing the paraspinal muscle FF, voxels in MRS or ROIs in LAVA-FLEX and IDEAL-IQ May have contained two adjacent muscles rather than one specific muscle. This was because when setting up the voxel for MRS, it was difficult to evaluate a single muscle considering the square shape of the voxel and the size of voxel to obtain a sufficient signal. The inclusion of fascial boundaries and intermuscular fat May have contributed to the heterogeneity in analysis in this study. Since the scan plane and the dog’s position cannot be entirely controlled during repeated scans, the optimal strategy to enhance repeatability May involve controlling the analysis of the ROI. In particular, LAVA-FLEX and IDEAL-IQ allow ROIs to be drawn freely regardless of their size and shape, making it easy to set an ROI for specific muscles. Further research focusing on repeatability using ROIs for specific muscles is needed.

When evaluating the FFs of the lumbar vertebrae and paraspinal muscles, it is important to know the physiological variance among healthy subjects. In humans, fatty infiltration can be affected by various factors such as the anatomical site, sex, age, obesity, and hormones; therefore, these factors should be considered when interpreting the FF (29–35). In this study, the FFs of the caudal lumbar vertebral bodies tended to be higher than those of the cranial lumbar spine in healthy dogs. Although there were no studies on fat content according to vertebral body location in dogs, the result of the present study was consistent with those obtained in human studies (29–31). A previous study showed increased FFs of the vertebral bodies from the first to the fifth lumbar vertebrae in healthy humans (29). In another study, a craniocaudal gradient of the vertebral FFs from T12 to L5 was observed in human patients without spinal bone disease (30). In human studies, centripetal bone marrow conversion has been suggested to be a cause of this lumbar vertebral FF gradient (29–32). In adult humans and dogs, the bone marrow converts from hematopoietic marrow to fatty marrow with a centripetal direction from the appendicular skeleton to the axial skeleton (31, 32). This centripetal conversion May contribute to the lumbar FF gradient. Another possible explanation is an indirect consequence of the increased mechanical stress on the caudal lumbar vertebrae (30). Since the sacropelvic complex is fixed, more force and stress are placed on the adjacent lower lumbar spine, which can cause higher fatty degeneration in the lower lumbar spine (30, 31).

Unlike the vertebral body FFs, there were no significant differences in the FFs of the paraspinal muscles according to the lumbar sites in this study. This result was not consistent with those from previous human studies, which showed that paravertebral muscle fatty infiltration generally increased from cranial to caudal, with the highest value at L5 (33–35). There are many possible causes for this discrepancy. First, relatively young dogs were included in this study. In humans, it was reported that lumbar paravertebral muscle fatty degeneration occurs relatively more slowly than vertebral body fatty degeneration with aging (33). Moreover, the younger the age of the individual, the less pronounced the difference in fatty degeneration of the paraspinal muscles according to the anatomical level (35). Therefore, we suggest that the age of dogs May have contributed to the lack of a FF gradient in the paraspinal muscles; however, due to the small number of subjects, differences by age could not be analyzed in this study. Second, the location of the muscles analyzed May have affected the results. In previous studies, fatty degeneration was most evident in the multifidus muscle in human patients with IVDD (33, 34). However, we did not include the multifidus muscle in this study because the cross-sectional area of this muscle was too small to place the voxel in it for MRS. Therefore, degenerative changes in the muscles May have been underestimated in this study. Unlike humans, dogs walk on all four limbs, so there May not be a difference in loading on the paravertebral muscles in the rear compared to the front like in humans. However, as far as author’s knowledge, there was no previous study to prove this.

In humans, many studies have been conducted to apply the FF of the vertebral bodies and paraspinal muscles in healthy subjects and patients with spinal diseases such as IVDD (2, 3, 8, 9, 12–16). These studies showed an association between the FF and histological IVDD grade, neurological symptom, and prognosis (2, 3, 8, 15). A recent study in dogs suggested that there is a correlation between fat degeneration and neurologic grade in dogs with IVDD and the potential use of fat degeneration as a biomarker in dogs with IVDD (7). In that study, paravertebral muscle fat fraction was not associated with the outcome of dogs with IVDD. However, due to the lack of studies in dogs, more data are needed in patients with IVDD. The results of this study suggest that LAVA-FLEX or IDEAL-IQ can be used as a routine sequence in dogs when obtaining spinal MRI in future studies to identify the association of fat infiltration in the vertebral bodies and paraspinal muscles with IVDD in dogs.

Although not included in the analysis in this study, LAVA-FLEX and IDEAL-IQ have several advantages for clinical use compared with MRS. First, these Dixon techniques are much faster for FF analysis, particularly when analyzing multiple sites. In this study, it took approximately 17 min for MRS to obtain the FFs of the vertebral bodies at each of six levels from L1–L6, whereas with LAVA-FLEX and IDEAL-IQ, it took less than 3 min. Second, LAVA-FLEX and IDEAL-IQ do not require post-processing compared with MRS, making them easy for clinicians to use. Third, MRS can only analyze the voxel set at the time of acquisition, while LAVA-FLEX and IDEAL-IQ allow setting and modification of the ROI in the obtained image. In addition, MRS requires the use of voxels of a certain size or a certain cuboid shape, but LAVA-FLEX and IDEAL-IQ can specify small ROIs with free shapes, allowing flexibility for evaluating specific anatomical muscles. Finally, LAVA-FLEX and IDEAL-IQ provide fat suppression images. When evaluating spinal disease, fat suppression images are necessary to distinguish the lesion from the epidural fat or paraspinal fat. Dixon techniques provide homogeneous and reliable fat separation and thus can be used as alternative sequences to other fat suppression images (16).

There were several limitations in this study. First, this study included only a small number of individuals. To overcome this limitation, analysis was performed at six different vertebral sites, but comparisons of more samples are needed through prospective studies in patients. Second, subjects included in this study were of the same breed and were similar in age and weight; thus, we could not consider various factors that can affect the FF. In humans, several factors including age, sex, and body weight can affect the FF; therefore, the FF in dogs could similarly be affected by various factors (29–35). However, these limitations did not pose a problem for the main purpose of this study, which was to evaluate the feasibility of LAVA-FLEX and IDEAL-IQ for assessing the FF compared with MRS. Further studies with larger numbers of subjects and wider range of variation in subject characteristics such as age, gender, body condition score, and breed are needed. Third, in this study, the FFs of the vertebral bodies and paraspinal muscles were not compared with histopathological findings. Previous human studies have showed a high correlation between Dixon-based or MRS-based FFs and histological results, but they have not been validated in dogs (35, 36). Although this study did not compare FFs to histological findings, we believe that the feasibility of LAVA-FLEX and IDEAL-IQ for evaluating the FFs of the lumbar vertebral bodies and paraspinal muscles in dogs was verified because it was compared with FFs derived from MRS, which is well-known as the gold standard method for FF evaluation.

## 5 Conclusion

In conclusion, the results of this study showed that LAVA-FLEX and IDEAL-IQ have high reproducibility and are highly correlated with MRS, the gold standard method, for measuring the FFs of the lumbar vertebral bodies and paraspinal muscles in dogs. In addition, the FFs can be obtained and analyzed much more easily and quickly with these methods compared with MRS. Therefore, we believe that LAVA-FLEX and IDEAL-IQ can be used as routine methods in spinal MRI in dogs.

## Data Availability

The datasets presented in this study can be found in online repositories. The names of the repository/repositories and accession number(s) can be found in the article/supplementary material.
